# Randomized, controlled pilot study of early rehabilitation strategies in acute respiratory failure

**DOI:** 10.1186/cc12478

**Published:** 2013-03-19

**Authors:** D Files, P Morris, S Shrestha, S Dhar, M Young, J Hauser, E Chmelo, C Thompson, L Dixon, K Murphy, B Nicklas, M Berry

**Affiliations:** 1 Wake Forest University School of Medicine, Winston Salem, NC, USA

## Introduction

Optimal patient evaluations of ICU rehabilitation therapy remain unclear.

## Methods

One hundred ICU patients with acute respiratory failure were randomized to receive early rehabilitation (ER) or usual-care (UC). Cohort 1 (*n *= 50) received ER as one physical therapy (PT) session/day versus UC; Cohort 2 (*n *= 50) received ER as 2 PT/day with the second session resistance training, versus UC. UC was without ER. Blood was drawn for cytokines through days 7. Cohort 2 underwent strength and physical functional assessments using the Short Physical Performance Battery (SPPB), a valid and reliable measure of physical function consisting of walking speed, balance, and repeated chair stands. It is a well-studied composite measure in older persons, but has not been used in ICU survivors. Small changes of 0.5 to 0.6 points in the SPPB have been shown to be clinically meaningful.

## Results

Baseline parameters were similar between groups. Median days from enrollment to first PT were 4 (IQR 1 to 7.25). Deaths occurred in eight UC subjects and four in ER (*P *= 0.22). For both arms, ventilator days, ICU days and hospital days were not statistically different. ER had ventilator-free days of 22, 95% CI = 19.9 to 24.6, where UC had 22.3 days, 19.9 to 24.6, *P *= 0.99. ICU-free days for ER was 21, 95% CI = 19.1 to 23.6, and that for UC was 21.0, 18.8 to 23.2, *P *= 0.84. Similarly, hospital days for ER was 16.7, 95% CI = 11.8 to 21.4, and for UC was 18.2, 13.8 to 22.7, *P *= 0.45. TNF, IL-6 and IL-8 through days 7 were not different between groups. Despite similar baseline acuity and inflammatory profiles, Cohort 2 ER group strength scores were numerically but not statistically higher. Grip strength, as a percentage of predicted for ER was 66 versus 39 for UC, *P *= 0.06. Dynamometry for ER was 211 versus 181 lbs for UC, *P *= 0.124. Although the difference in SPPB values for ER versus UC (5 vs. 3, *P *= 0.172) was not statistically different, it was greater than the minimal clinically significant difference. There were no differences in adverse events.

## Conclusion

In this pilot study, early ICU rehabilitation was safe, and was associated with numerically although not statistically shorter hospital stay, greater strength and improved functional scores. Particularly, the SPPB demonstrated discriminatory ability in groups of ICU survivors with low physical function. Future early ICU rehabilitation studies should consider ICU survivor assessments using the SPPB due to its ease, reproducibility and discriminatory ability following ICU and hospital discharge.

